# Lactylation: a promising target for musculoskeletal disorders via interactions with chronic inflammation^☆^

**DOI:** 10.1016/j.jare.2025.09.058

**Published:** 2025-10-08

**Authors:** Tiantian Wang, Sihan Chen, Zhen Hong

**Affiliations:** a Department of Neurology, Institute of Neurology and Disease, West China Hospital of Sichuan University, Chengdu, Sichuan, PR China; b Institute of Brain Science and Brain-inspired Technology of West China Hospital, Sichuan University, Chengdu, Sichuan, PR China; c Department of Neurology, Chengdu Shangjin Nanfu Hospital, Chengdu, Sichuan, PR China; d West China School of Nursing, Sichuan University, Chengdu, Sichuan, PR China

**Keywords:** Lactylation, SPM, Chronic inflammation, Musculoskeletal disorders, Macrophage

## Abstract

Musculoskeletal disorders (MSDs) encompass conditions that affect bones, joints, and muscles, leading to substantial pain and functional impairment and representing a major global health concern. In MSDs, key metabolic processes—such as glycolysis and lactate accumulation under hypoxic conditions—disrupt cellular energy homeostasis. Lactylation, a post-translational modification arising from excessive lactate, modifies both histone and non-histone proteins and has been implicated in inflammation and MSD pathogenesis. Preclinical studies indicate that targeting lactylation holds promise for the treatment of various MSDs. Systemic, chronic, low-grade inflammation (SCLGI) is thought to contribute to both the onset and persistence of many MSDs, with its unresolved state driving chronic disease progression. Promoting the resolution of SCLGI may alleviate symptoms, particularly through specialized pro-resolving mediators (SPMs) derived from dietary essential polyunsaturated fatty acids (PUFAs). Both SPMs and their small-molecule analogs have demonstrated therapeutic benefits in animal models of inflammation-related disorders, including arthropathies, osteoporosis, and muscular dystrophy.

This review proposes a novel therapeutic strategy for MSDs that integrates lactylation inhibitors or agonists with agents that facilitate SCLGI resolution. Such a combined approach may enhance the effectiveness of MSD management.

## Highlights

• MSDs are defined by the inability to resolve chronic inflammation.
• Specialized pro-resolving mediators (SPMs) reduce the symptoms of MSDs.
• Lactylation plays a role in MSD.

## Graphical abstract

**Figure:**
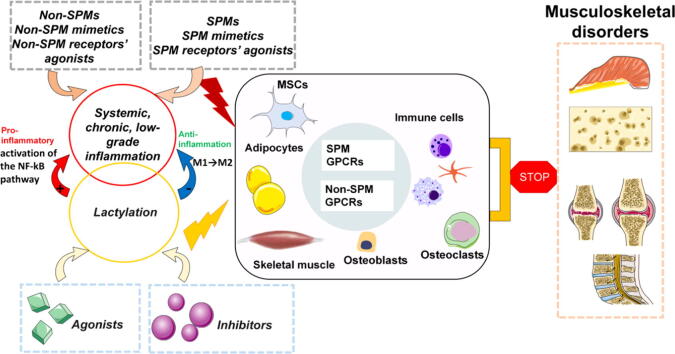

## Introduction

Musculoskeletal disorders (MSDs) comprise a broad spectrum of degenerative conditions affecting bones, joints, muscles, and connective tissues. These disorders represent a leading global cause of chronic pain and disability, resulting in substantial socioeconomic burdens. As global populations age and life expectancy increases, MSD prevalence continues to rise. According to the 2019 Global Burden of Disease (GBD) study, approximately 1.71 billion people worldwide suffer from MSDs [1].

The prevailing theory suggests that Systemic, chronic, low-grade inflammation (SCLGI) plays a central role in the development and persistence of various age-related chronic diseases, including MSDs [[2], [3], [4], [5], [6], [7]]. Nevertheless, therapeutic interventions that directly target inflammatory pathways often achieve only limited efficacy in these conditions. For example, prolonged systemic inhibition of nuclear factor κB (NF-κB) is hindered by potential adverse effects, and selective cytokine blockade—such as interleukin-1β(IL-1β) targeting—has proven ineffective in osteoarthritis (OA) because it fails to address other inflammatory mediators [8]. The inability to resolve SCLGI perpetuates chronic inflammation, indicating that successful resolution could alleviate symptoms in SCLGI-associated disorders. specialized pro-resolving mediators (SPMs), biosynthesized from essential dietary polyunsaturated fatty acids (PUFAs), can facilitate this resolution [9]. SPMs elicit cell-specific responses by engaging G protein–coupled receptors (GPCRs) [10]. Furthermore, activation of non-SPM GPCRs in adipocytes, osteoblasts, immune cells, and muscle cells under SCLGI conditions may also attenuate inflammation and MSD pathology. Evidence from animal models demonstrates that PUFAs, SPMs, non-SPMs, their synthetic analogs, and receptor agonists exert beneficial effects in inflammation-related disorders, including arthropathies, osteoporosis, and muscular dystrophy [[11], [12], [13]]. However, research exploring the interplay between SPMs, non-SPMs, and MSDs remains at an early stage, and direct clinical evidence in humans is scarce. Therefore, further investigation into additional mechanisms underlying chronic inflammation is essential for advancing translational applications.

In recent years, lactate metabolism and its downstream epigenetic effects have gained recognition as critical regulators of inflammatory responses. Once regarded merely as a metabolic byproduct, lactate is now understood to function as a key signaling molecule in intercellular communication, immune modulation, and tumor biology [[14], [15], [16], [17], [18]]. Importantly, lactate can accumulate within hypoxic or metabolically stressed microenvironments—such as those found in degenerating musculoskeletal tissues—where it may modulate inflammation through epigenetic mechanisms.

A landmark discovery in 2019 demonstrated that lactate serves as a precursor for lysine lactylation (Kla), a novel post-translational modification occurring on both histone and non-histone proteins [14,19,20]. Classification into histone or non-histone lactylation depends on the identity of the modified protein. Similar to methylation and acetylation, histone lactylation is regulated by “readers” (proteins that recognize lactylation and mediate specific functions), “writers” (lactyltransferases), and “erasers” (delactylases) [14,20]. Accumulating evidence links lactylation to the progression of multiple diseases, with lactate accumulation correlating with pathological development. Both histone and non-histone lactylation are integral to key cellular functions and disease-related processes, albeit via distinct regulatory pathways. Histone lactylation primarily affects core histones such as H3 and H4, influencing disease mechanisms mainly through the actions of histone deacetylases (HDACs) and histone acetyltransferases (HATs), which remodel chromatin structure and function. In contrast, non-histone lactylation modifies diverse proteins, including metabolic enzymes, transcriptional regulators, and signaling molecules. This modification influences protein stability, protein–protein interactions, and enzymatic activity, often involving enzymes such as lactate dehydrogenase (LDH), pyruvate kinase (PK), and glycerate kinase (GK) [18,21] (Fig. 1a).

**Fig. 1a:**
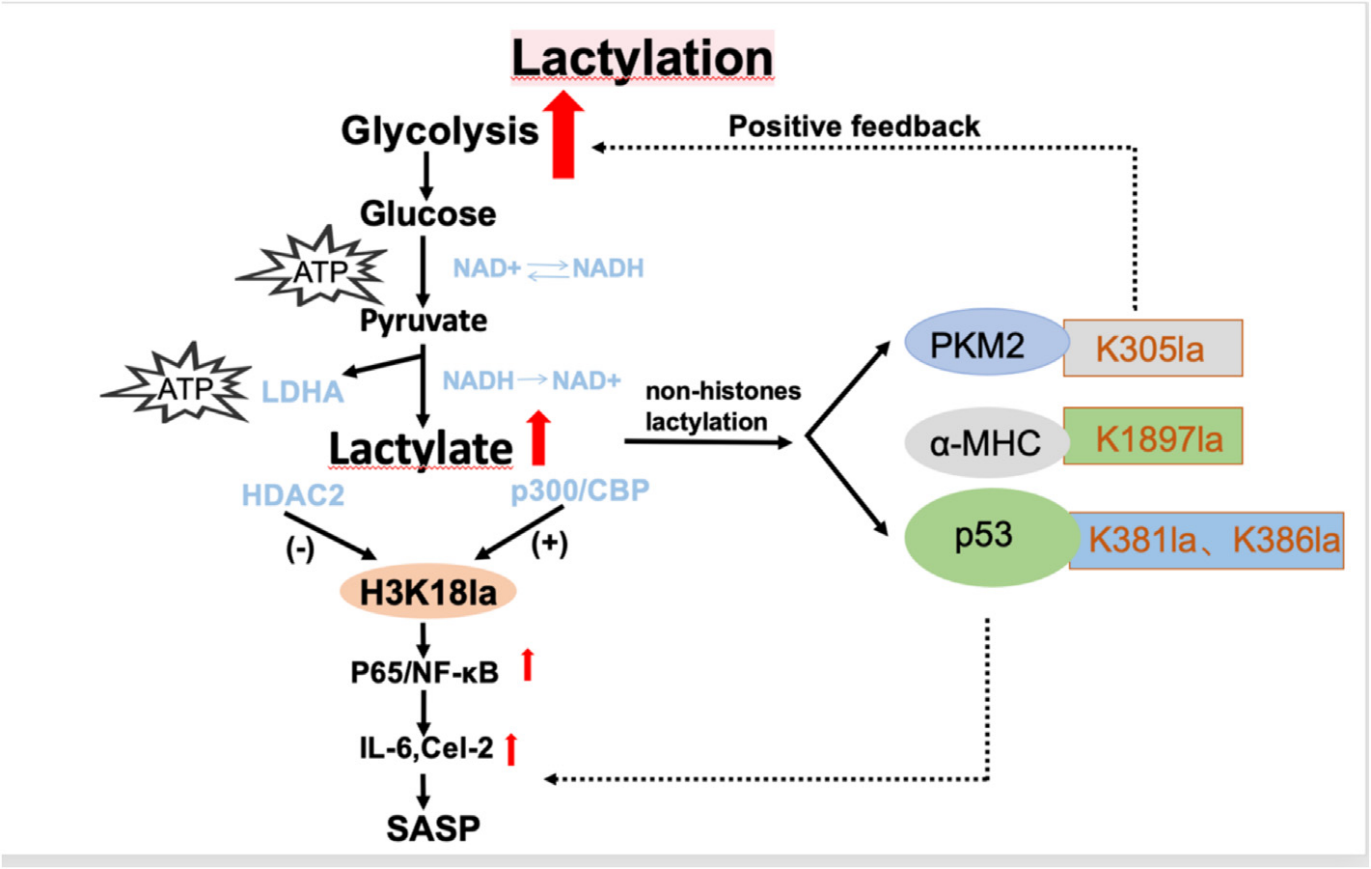
Metabolic map of lactylation.

Emerging evidence indicates a strong association between lactylation and inflammatory processes. For example, histone H4 lysine 12 lactylation (H4K12la) has been shown to exacerbate inflammation by activating the NF-κB signaling pathway [22]. Furthermore, both lactate production and the levels of specific histone lactylation marks—H4K12la and H4 lysine 5 lactylation (H4K5la)—are elevated in a phosphofructokinase-2/fructose-2,6-bisphosphatase 3 (PFKFB3)-dependent manner in ischemia–reperfusion injury mouse models and in vitro systems. Increased H4K12la is enriched at promoter regions of certain NF-κB pathway components, thereby promoting their transcription and amplifying NF-κB signaling [23]. Pharmacological modulation of lactylation has demonstrated therapeutic potential in preclinical inflammation models [24], highlighting its promise as a novel anti-inflammatory strategy.

Recent studies indicate that lactate accumulation within the MSD microenvironment contributes to the pathogenesis of conditions such as intervertebral disc degeneration (IVDD) [25], osteoporosis [26,27], rheumatoid arthritis (RA) [28,29], as well as osteoarthritis (OA) [30]. However, the underlying mechanisms remain incompletely understood. Given lactate’s dual role as both a metabolic intermediate and an inflammatory signal, modulating lactylation—through either inhibition or activation—may represent a promising therapeutic approach. Moreover, combining lactylation-targeted interventions with SCLGI-resolving agents, such as SPMs or GPCR agonists, could produce synergistic effects. This review synthesizes current advances in understanding lactylation within the context of chronic inflammation and MSDs, and proposes that integrating lactylation-targeted strategies with inflammation-resolving pathways could yield novel therapeutic avenues for OA, RA, IVDD, osteoporosis, and sarcopenia (Fig. 1b). This integrative hypothesis forms the central theme of our discussion.

**Fig. 1b:**
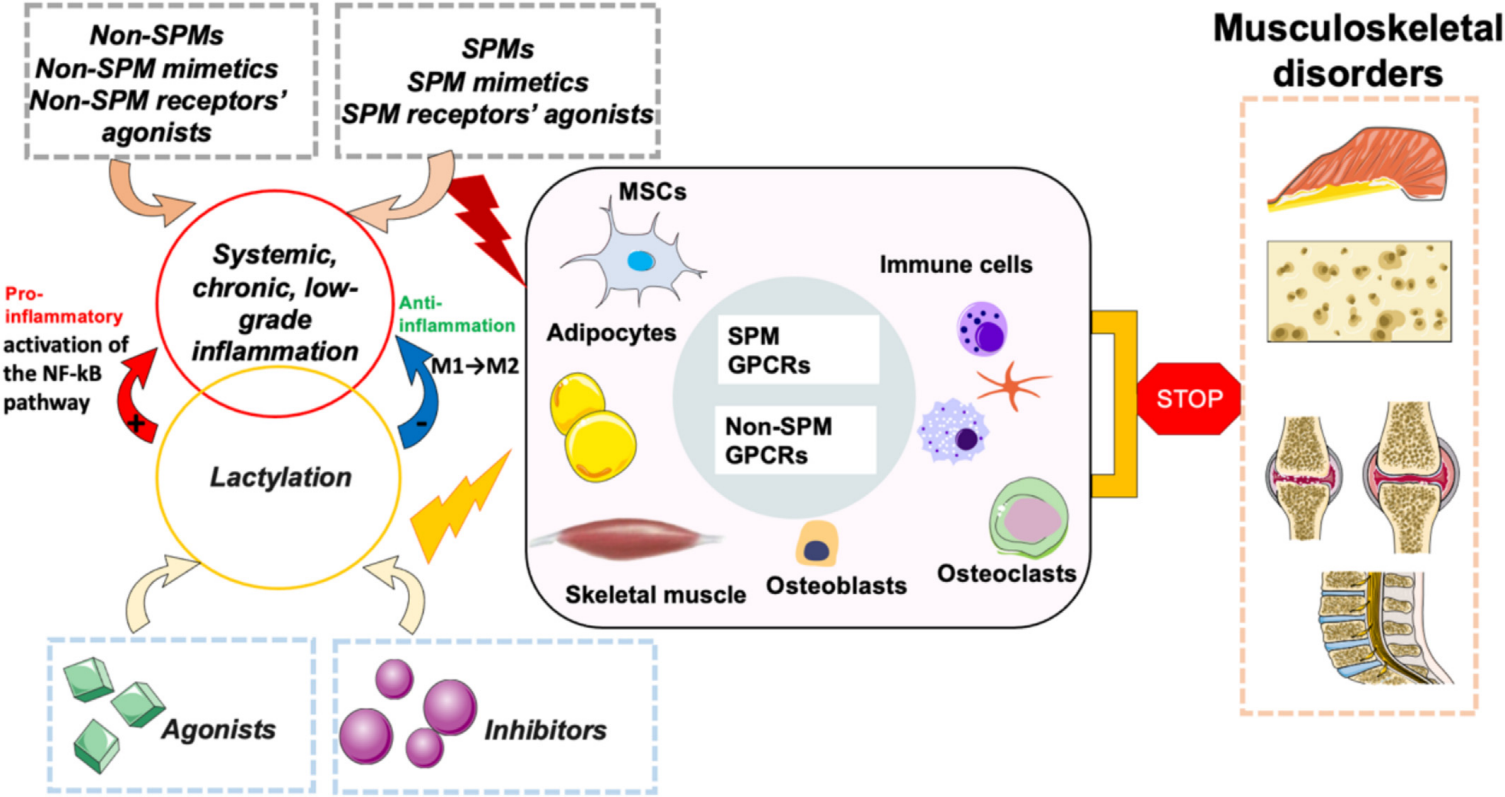
Novel anti-MSDs strategies Given its significant impact on the production of pro-inflammatory cytokines, research is actively exploring compounds that can activate or inhibit lactylation. Finally, this review investigates the potential of lactylation inhibitors and agonists in the context of MSDs and posits that targeting lactylation in conjunction with SCLGI-resolving compounds could provide a promising therapeutic strategy for conditions such as IVDD, OA, osteoporosis, and sarcopenia (Fig. 1b).

## The dual roles of lactylation in the progression of inflammation in musculoskeletal disorders？

Recent studies have underscored the pivotal role of modulating macrophage responses along the inflammatory-to-reparative spectrum in diverse inflammatory diseases, including atherosclerosis, obesity, cancer, and colitis, where shifts in macrophage polarization profoundly affect disease progression and outcomes [31,32]. Macrophages are broadly categorized into two functional phenotypes: pro-inflammatory M1 and anti-inflammatory M2. Successful resolution of inflammation requires a timely transition from the M1 to the M2 state, characterized by the suppression of inflammatory cytokines, the activation of reparative gene programs such as Arg1, and a metabolic reprogramming from glycolysis toward oxidative phosphorylation [33].

Emerging evidence indicates that lactate accumulation—a common feature of metabolically active or hypoxic tissues—is a key determinant of histone lactylation, a recently identified epigenetic modification essential for regulating macrophage phenotype [21]. Histone lactylation has been shown to promote the phenotypic transition of macrophages from the pro-inflammatory M1 state to the anti-inflammatory M2 state, particularly in metabolically stressed or reparative tissues. For example, Zhang et al. demonstrated that both lactate and histone lactylation drive this transition in adipose tissue macrophages [21]. Irizarry-C et al. further reported that macrophages lacking the B-cell adaptor for PI3K (BCAP) exhibit reduced lactate production, diminished histone lactylation, and impaired expression of reparative genes. Remarkably, restoring lactylation via N-acetyl-lactylamide (Nala) or exogenous lactate reinstated Arg1 and Klf4 expression within 12 h in BCAP^−/− bone marrow-derived macrophages, underscoring lactylation as a critical regulator of macrophage repair functions [34]. Additional studies have linked histone lactylation to metabolic reprogramming during inflammatory responses. In particular, Arg1 expression in M2 macrophages correlates with elevated histone lactylation levels and is associated with interleukin-6(IL-6)– and Arg1-dependent metabolic rewiring [35,36]. Recent findings also identify mitochondrial fragmentation—a hallmark of M2 polarization—as a driver of lactate production and subsequent histone lactylation. Collectively, these insights highlight the intricate interplay between mitochondrial dynamics, metabolic state, and epigenetic regulation in orchestrating macrophage plasticity and maintaining immune homeostasis [37].

In MSDs—including osteoporosis, OA, RA, IVDD, and sarcopenia—persistent low-grade inflammation and metabolic dysregulation are hallmark features. Elevated extracellular lactate levels are frequently detected in these conditions and are closely linked to aberrant histone lactylation within tissue-resident immune cells, particularly macrophages. In the following sections, we focus on how this conserved lactate–lactylation–macrophage axis is modulated or disrupted across different MSD contexts, shaping distinct pathological trajectories. Aerobic glycolysis–driven lactate production promotes histone lactylation, which subsequently reprograms gene expression toward anti-inflammatory or tissue-repair phenotypes. Key mediators in this process include monocarboxylate transporter 4 (MCT4), which regulates lactate efflux, and p300 acetyltransferase, which catalyzes lactylation of histone residues such as H3 lysine 18 lactylation (H3K18la) [38]. Interestingly, while polarization toward the M1 phenotype is accompanied by increased histone lactylation, the subsequent shift to M2 is associated with reduced lactylation, suggesting a temporally dynamic regulatory role [38].

Despite substantial evidence supporting a pro-resolving role for lactylation, accumulating data indicate that its effects are highly context-dependent and may, in certain settings, promote rather than resolve inflammation. For example, Chu et al. reported that elevated H3K18la levels in patients with septic shock correlated with increased expression of both inflammatory markers and Arg1, suggesting that lactylation may serve as a biomarker of immune dysregulation rather than a mediator of resolution [39]. Despite substantial evidence supporting a pro-resolving role for lactylation, accumulating data indicate that its effects are highly context-dependent and may, in certain settings, promote rather than resolve inflammation. For example, Chu et al. reported that elevated H3K18la levels in patients with septic shock correlated with increased expression of both inflammatory markers and Arg1, suggesting that lactylation may serve as a biomarker of immune dysregulation rather than a mediator of resolution [40]. Similarly, in neuroinflammatory contexts—such as aging microglia and senescent glial cells from Alzheimer’s disease (AD) models—high H3K18la levels have been observed. Integrative ChIP-qPCR and RNA-seq analyses revealed that lactate-induced histone lactylation increases NF-κB1 (p50) promoter occupancy, thereby driving transcription of senescence-associated secretory phenotype (SASP) cytokines including IL-6 and interleukin-8(IL-8) [41,42]. Collectively, these findings underscore that lactylation is not universally beneficial; under certain pathological conditions, it may exacerbate chronic inflammation and accelerate tissue degeneration.

Taken together, these findings support a nuanced understanding of histone lactylation in inflammation: although it often promotes anti-inflammatory and reparative gene programs, it can, under conditions of prolonged metabolic stress or unresolved inflammation, trigger pathogenic pathways that exacerbate inflammatory responses. This context-dependent duality is particularly pertinent to MSDs, which are characterized by SCLGI and dynamic metabolic fluctuations. Across conditions such as osteoporosis, OA, RA, IVDD, and sarcopenia, common pathogenic threads emerge, including dysregulated macrophage polarization, aberrant activation of the NF-κB pathway, and metabolic–epigenetic feedback loops driven by lactate accumulation and histone lactylation. Recognizing these shared mechanisms allows for a unified conceptual framework that can be tailored to the specific tissue microenvironment in each disorder. We propose that, in MSDs, lactylation serves as a pivotal metabolic–epigenetic interface that shapes the trajectory of inflammatory regulation.

## Causal or Correlative? exploring the relationship between lactylation and SCLGI

Although current studies strongly suggest a correlation between elevated lactate/lactylation levels and the persistence of SCLGI in MSDs, the directionality of this relationship remains unclear. Most available evidence is correlative, and definitive causal links have yet to be demonstrated in vivo. Nonetheless, emerging preclinical models and mechanistic hypotheses now provide a framework for testing bidirectional interactions between lactate metabolism, lactylation, and chronic inflammation.

On one hand, lactate accumulation under chronic metabolic stress is a well-recognized feature of SCLGI environments. Elevated lactate can drive histone lactylation—particularly at regulatory sites such as H3K18la—thereby activating transcriptional programs that may either promote inflammation resolution (e.g., Arg1, Klf4) or, in pathological contexts, exacerbate inflammation via NF-κB pathway activation. For instance, L-lactylation (L-Kla) facilitates macrophage polarization toward an anti-inflammatory M2 phenotype [21], whereas D-lactylation (D-Kla) has been linked to increased production of inflammatory cytokines in macrophages [43]. Similarly, in senescent microglia and synovial fibroblasts, H3K18la enhances the expression of IL-6, IL-8, and MMPs, supporting a model in which lactylation can reinforce SCLGI under specific conditions.

Conversely, persistent SCLGI may itself drive aberrant lactylation. Chronic immune cell activation sustains glycolytic flux and lactate production, expanding the substrate pool for histone lactylation [44]. In this scenario, lactylation functions downstream of unresolved inflammation, locking cells into a maladaptive metabolic–epigenetic state. Establishing causality will require future studies to adopt the following strategies:

In vitro models: Apply lactylation agonists or inhibitors (e.g., p300 inhibitors, Lactate dehydrogenase A(LDHA) modulators) to primary macrophages or fibroblasts cultured under defined inflammatory conditions, and assess resulting changes in cytokine secretion profiles and genome-wide epigenetic signatures.

In vivo models: Utilize transgenic mice with immune cell–specific deletion or overexpression of lactylation “writers” (e.g., p300) or “erasers” (e.g., sirtuin 3 (SIRT3)), and evaluate outcomes in SCLGI-driven disease models such as osteoarthritis (DMM model), RA (CIA model), or aging-related sarcopenia.

Time-resolved studies: Determine whether lactylation precedes or follows SCLGI onset by combining metabolic flux analyses with chromatin immunoprecipitation-sequencing (ChIP-seq) profiling of lactylated histone marks across defined disease stages.

In the absence of definitive causal evidence, our working model posits that lactylation and SCLGI form a self-reinforcing feedback loop: metabolic dysfunction drives lactylation, which subsequently reprograms immune gene expression to sustain chronic inflammation. Therapeutic strategies aimed at disrupting this loop from both the metabolic and epigenetic sides may offer a novel and effective approach for halting disease progression in MSDs.

## Novel epigenetic roles of lactylation in osteoporosis

Osteoporosis is a systemic, age-related skeletal disorder characterized by reduced bone mass and deterioration of bone microarchitecture, resulting in increased fracture risk [45,46]. It encompasses both senile and postmenopausal subtypes. Bone homeostasis is maintained through the coordinated activities of osteoblasts, osteoclasts, and osteocytes. Osteoblasts, derived from mesenchymal stem cells that can also differentiate into chondrocytes, adipocytes, and fibroblasts, rely on key transcription factors such as Runx2 and Osterix for lineage commitment. In contrast, osteoclasts originate from hematopoietic stem cells and require receptor activator of NF-κB ligand (RANKL) and macrophage colony-stimulating factor (M−CSF) for maturation. Estrogen deficiency disrupts this balance by stimulating osteoblasts to secrete factors including IL-6, Fas ligand(FasL), and RANKL, which enhance bone resorption, while concurrently reducing osteoprotegerin (OPG) production, thereby facilitating osteoclastogenesis. Moreover, estrogen depletion accelerates cellular senescence in osteoblasts, osteoclasts, and osteocytes, further exacerbating bone loss.

Numerous studies have demonstrated that inflammatory conditions contribute to osteoporosis risk, highlighting the pivotal role of SCLGI in its pathogenesis. Inflammatory cytokines secreted by immune cells—particularly macrophages—affect bone remodeling by stimulating osteoblasts to produce the osteoclast differentiation-inducing factor RANKL (TNFSF11), thereby promoting osteoclastogenesis. These cytokines can also act directly on osteoclasts to enhance bone resorption. In osteoporosis, histone lactylation may exert dual effects: (i) mitigating inflammation by regulating macrophage polarization and suppressing pro-inflammatory factor expression; and (ii) supporting bone homeostasis by promoting osteogenic differentiation and reducing bone tissue damage. Together, these observations suggest a mechanistic interplay between SCLGI and lactylation in the development and progression of osteoporosis.

Previous research has highlighted the role of histone lactylation in osteoblast differentiation [47]. Nian et al. reported that during the mineralization of the pre-osteoblast cell line MC3T3-E1, both intracellular lactate levels and histone lactylation increased, correlating with elevated expression of lactate dehydrogenase A [48]. ChIP-qPCR analysis revealed that lactylation of H3K18la is associated with the transcriptional activation of activator protein-1 (AP-1). Moreover, increased H3K18la within the promoter region of the *JunB* gene positively regulates osteoblast differentiation. Another study demonstrated that intracellular lactate levels and pan-histone lactylation (Pan-Kla) are positively correlated with bone morphogenetic protein 2 (BMP-2)-induced osteoblast differentiation, with p300 acting as the primary mediator of this modification. These findings suggest that lactate derived from glucose metabolism can enhance histone lactylation via p300, thereby modulating gene expression programs critical for osteoblast differentiation. Notably, lactate produced by endothelial cells (ECs) can induce histone lactylation in bone marrow mesenchymal stem cells (BMSCs), promoting osteogenesis [26]. Consistent with Nian’s findings, lactate was found to primarily influence lactylation at the H3K18 site. In ovariectomized (OVX) mice—a widely used model of osteoporosis—reduced expression of pyruvate kinase M2 (PKM2), a key enzyme in endothelial glycolysis, was associated with diminished bone vessel density. Endothelial cell-specific PKM2-deficient mice exhibited lower H3K18la levels in BMSCs and worsened osteoporotic phenotypes. Remarkably, both OVX and *Pkm*2ΔEC mice showed improved bone parameters following lactate infusion or exercise, which elevated serum lactate levels and restored H3K18la in BMSCs, thereby promoting osteoblast differentiation. Furthermore, patients with osteoporosis displayed reduced serum lactate concentrations and lower H3K18la in BMSCs. Functional analysis indicated that H3K18la regulates key osteogenesis-related genes, including *COL1A2*, cartilage oligomeric matrix protein (COMP), ectonucleotide pyrophosphatase/phosphodiesterase 1 (ENPP1), and transcription factor 7-like 2(TCF7L2*)* [26].

In osteoporosis, macrophages—particularly the balance between their pro-inflammatory M1 and anti-inflammatory M2 phenotypes—play a pivotal role in osteoclastogenesis and bone remodeling. OVX mouse models exhibit an increased M1/M2 ratio, which contributes to postmenopausal bone loss [49]. Within this framework, the conserved lactate–lactylation axis modulates the phenotypic polarization of bone marrow-derived macrophages: lactylation of PKM2 attenuates glycolysis and promotes reparative M2 characteristics. In RAW264.7 cells, lactate-induced histone lactylation suppresses the expression of pro-inflammatory cytokines IL-1β, IL-6, and tumor necrosis factor α (TNF-α), while enhancing the expression of M2 markers Arg1 and CD206; these effects are reversed by the p300 inhibitor C646 [50,51]. These findings underscore the therapeutic potential of modulating lactylation to restore macrophage polarization and mitigate osteoclast-mediated bone loss. Collectively, the evidence indicates that lactylation plays a critical role in regulating macrophage polarization and inhibiting the secretion of inflammatory mediators implicated in osteoporosis. In addition, lactylation promotes osteogenic differentiation, reduces bone damage, and slows osteoporosis progression. Physiological conditions that increase lactate production—such as exercise—can enhance histone lactylation, thereby suppressing inflammatory responses and potentially regulating bone remodeling.

## The lactylation in OA

OA is a complex, multifactorial disease and the most prevalent form of arthritis, with an etiology that remains incompletely understood. It induces pathological alterations across all joint tissues, including cartilage, subchondral bone, ligaments, menisci, the joint capsule, and the synovial membrane. The progression of OA is predominantly driven by catabolic mediators released by articular chondrocytes in response to biomechanical and inflammatory stimuli. Among the molecular pathways implicated, the chronic activation of NF-κB has emerged as a central contributor to OA pathogenesis. Chondrocytes can be activated by mechanical overload, injury, and inflammatory cytokines associated with aging and metabolic disorders, initiating a cascade of catabolic events. Progressive cartilage degradation amplifies mechanical stress and joint damage, which in turn heightens inflammatory signaling within the synovial space and sustains NF-κB activation in a self-perpetuating cycle. However, systemic and sustained inhibition of NF-κB in humans remains challenging due to potential adverse effects. Likewise, biologic therapies targeting individual cytokines—such as IL-1β—have shown limited efficacy in OA, as they fail to address the broad spectrum of inflammatory drivers involved.

Recent studies have revealed a bidirectional regulatory interplay between inflammation and metabolism. In OA animal models and lipopolysaccharide (LPS)-stimulated cell cultures, total Pan-Kla and histone H3K18la levels were markedly elevated compared with normal controls [30]. LDHA is a key enzyme in lactate production and plays a pivotal role in modulating chondrocyte function during OA, in part by regulating reactive oxygen species (ROS) generation [[52], [53], [54]]. Silencing LDHA in LPS-stimulated chondrocytes reversed the decline in type II collagen (Col2) levels, reduced matrix metalloproteinase-13 (MMP13) expression, and decreased both glucose consumption and lactate production. In vivo, LDHA knockdown mitigated cartilage degeneration, lowered Osteoarthritis Research Society International (OARSI) scores, preserved proteoglycan content, and reduced cartilage thinning and chondrocyte loss, thereby facilitating cartilage repair in OA mouse models. Mechanistically, upregulation of LDHA enhances lactate production, which in turn promotes histone lactylation. LDHA thus functions as a mediator of lactylation, influencing the histone lactylation status of target genes [48]. For example, hypoxic conditions suppress LDHA activity, leading to reduced lactate levels and diminished histone lactylation. Consistently, LDHA knockdown significantly decreased histone lactylation in oxygen–glucose deprivation/reoxygenation (OGD/R)-treated N2a cells, further implicating LDHA in OA pathogenesis through its regulatory effect on histone lactylation [55]. From a translational perspective, developing small-molecule inhibitors or modulators that either target the histone acetyltransferase-like activity of LDHA or stabilize the H3K18ac mark at the TPI1 locus may offer a promising therapeutic avenue.

In OA, macrophage polarization is a key determinant of inflammatory modulation and cartilage repair. MSCs contribute to cartilage regeneration by suppressing pro-inflammatory M1 polarization and promoting anti-inflammatory M2 polarization. Osteoclasts—derived from the monocyte–macrophage lineage—are also implicated in OA-related pain. Symptomatic OA patients show elevated levels of bone resorption markers, such as tartrate-resistant acid phosphatase 5b (TRAP5b), and increased densities of tartrate-resistant acid phosphatase (TRAP)-positive osteoclasts in the subchondral bone compared with asymptomatic individuals. Patients experiencing OA pain who undergo total knee arthroplasty exhibit higher numbers of osteoclast precursors in subchondral bone expressing nerve growth factor (NGF), a mediator of inflammatory pain. Synovitis further exacerbates osteoclast activation in OA, as TNFα synergistically amplifies receptor activator of nuclear factor κB (RANK) and RANKL signaling together with cytokines such as interleukin-1 (IL-1). TNFα enhances RANK expression on osteoclast precursors, stimulates RANKL production by osteoblasts and stromal cells, and promotes the fusion and migration of osteoclast precursors to inflamed sites. In line with findings from other MSDs, lactate-induced histone lactylation facilitates the M1-to-M2 transition, potentially supporting tissue regeneration.

In summary, although direct evidence connecting lactylation to OA remains limited, emerging studies suggest that it plays a pivotal role in disease pathogenesis, potentially through the modulation of chronic inflammation and cellular energy metabolism.

## The role of lactylation in RA

RA is a chronic autoimmune disorder characterized by persistent synovitis and progressive joint destruction, leading to chronic pain, bone erosion, and disability. Affecting approximately 0.5–1 % of the global population, it ranks among the most prevalent chronic inflammatory diseases. The autoimmune response in RA is mediated by autoreactive T cells, pro-inflammatory synovial macrophages, and B cells that produce autoantibodies. In patients with RA, peripheral blood mononuclear cells (PBMCs) exhibit DNA damage resulting from disrupted chromatin organization and impaired DNA repair. Synovial macrophages further aggravate the disease by secreting pro-inflammatory cytokines [56].

Lactate, a byproduct of glycolysis, has emerged as a critical factor in RA, exerting profound effects on cellular function through its accumulation in the synovium [57]. In fibroblast-like synoviocytes (FLSs), D-lactate activates hypoxia-inducible factor 1-alpha (HIF-1α) via the phosphatidylinositol 3-kinase (PI3K)/Akt/ NF-κB signaling pathway. In RA-FLSs, lactate enhances glycolysis, migration, invasion, and IL-6 expression [58]. HIF-1α activation, in turn, promotes IL-6 transcription and upregulates GLUT1 and L-LDHA, channeling glucose metabolism toward glycolysis. Targeting RA-FLSs therefore represents a promising strategy to attenuate RA progression. In addition, histone lysine lactylation has emerged as a novel epigenetic modification that promotes gene transcription, making it a potential therapeutic target in RA. The natural compound artemisinin (ART), derived from *Artemisia annua* L., has been shown to enhance the interaction between p300 and PKM2, thereby promoting PKM2 lactylation. This modification inhibits RA-FLS proliferation by preventing PKM2 nuclear translocation, ultimately alleviating RA pathology.

In RA, the lactate–lactylation axis modulates not only macrophages but also a range of immune and stromal cells. While elevated histone lactylation enhances M2-associated gene expression in macrophages, RA synovial FLSs import lactate via monocarboxylate transporter 1 (MCT1), thereby acquiring a pro-inflammatory phenotype. Lactate also impacts other immune cell subsets; for example, it impairs dendritic cell maturation in response to LPS stimulation. In CD4^+^ and CD8^+^ T cells, lactate is transported through SLC5A12 and MCT1, respectively, triggering a “stop migration” signal that suppresses glycolysis while promoting fatty acid oxidation, effectively retaining these cells at sites of inflammation. Moreover, lactate drives a phenotypic shift in CD4^+^ T cells toward Th17 lineage, resulting in elevated interleukin-17(LPS17) production and exacerbating inflammatory responses. Collectively, these findings highlight the central role of lactate/lactylation pathways in orchestrating both innate and adaptive immune mechanisms in RA pathogenesis.

Integrating single-cell RNA sequencing (scRNA-seq) with advanced bioinformatics and machine learning has revealed key lactylation-associated genes—such as *NDUFB3*, *NGLY1*, and *SLC25A4*—as potential biomarkers for RA. Plasma cells with elevated lactylation scores exhibit enhanced metabolic activity, including both oxidative phosphorylation and glycolysis, to sustain the high energy demands of antibody production. A strong positive correlation between the RA lactylation score and immune cell infiltration—including Tregs, Th1 and Th2 cells, B cells, and NK cells—suggests that plasma cell lactylation may contribute to the pro-inflammatory milieu of RA. These observations are consistent with emerging evidence underscoring the role of metabolic reprogramming in immune-mediated inflammation. Therapeutically, targeting lactylation pathways in plasma cells offers a promising strategy to reprogram their metabolism, reduce autoantibody production, and attenuate inflammatory responses in RA.

In conclusion, current evidence highlights the pivotal role of lactylation in the pathogenesis of RA, offering novel insights into its underlying metabolic and immunological mechanisms. These findings not only identify potential biomarkers but also point to promising therapeutic targets for this debilitating condition. Future research should aim to elucidate the precise molecular pathways through which lactylation regulates immune responses and cellular metabolism. Moreover, large-scale patient cohort studies are warranted to validate these observations and to assess the clinical potential of targeting lactylation pathways in RA management.

## The lactylation in IVDD

IVDD is a major contributor to low back pain. The intervertebral disc consists of two primary components: the inner nucleus pulposus (NP), rich in glycosaminoglycans, and the outer fibrocartilaginous annulus fibrosus (AF), composed of concentric layers of collagen that encase the NP. The disc is anchored to adjacent vertebrae by the superior and inferior cartilaginous endplates (EPs), which are essential for nutrient exchange between the vascularized vertebrae and the avascular disc. Nucleus pulposus cells are responsible for synthesizing extracellular matrix (ECM) proteins, including proteoglycans and type II collagen. The elastic and colloidal properties of these cells are critical for preserving disc height and dissipating axial mechanical stress. The progression of IVDD is driven by multiple processes, including inflammation, cellular senescence, apoptosis, and pyroptosis—a pro-inflammatory form of programmed cell death—in NP cells [59,60].

The intervertebral disc (IVD) is the largest avascular structure in the human body, entirely lacking blood vessels. This absence of vascularization creates a hypoxic microenvironment within the NP, where oxygen concentrations can drop to as low as 1 % [61]. Nucleus pulposus cells (NPCs) rely primarily on glycolysis for energy production, leading to lactate accumulation. This, in turn, elevates lactic acid levels and contributes to a relatively low intracellular pH [62].

Lactate, a key byproduct of glycolysis, is strongly linked to the pathogenesis of IVDD. Elevated lactate concentrations promote a shift toward catabolic pathways while suppressing anabolic activities in acid-sensitive NPCs, ultimately driving cellular senescence and ECM degradation [63]. Bartels et al. reported that lactate levels in patients with back pain peak between 2 and 6 mM, creating an acidic microenvironment that compromises cell viability [64]. Similarly, Ohshima and Urban observed that increased lactate concentrations inhibit proteoglycan synthesis, thereby reducing proteoglycan content and contributing to disc degeneration [63]. In vitro, Wu et al. demonstrated that NPCs exposed to 6 mM lactate underwent apoptosis, whereas exposure to 2 mM lactate did not induce cell death and even enhanced type II collagen expression [65]. In contrast, high lactate levels (6 mM) increased type I collagen expression. Collectively, these findings suggest that lower lactate concentrations may preserve Nucleus pulposus cell (NPC) function, while higher levels hinder tissue repair. Targeting glycolytic reprogramming in NPCs could therefore represent a promising strategy to maintain or restore disc integrity and slow IVDD progression.

Previous research has established a link between IVDD and increased glycolysis with consequent lactate accumulation within the IVD, which may impair repair and regeneration of NPCs [[66], [67], [68]]. This suggests that lactylation and its associated genes or pathways may significantly influence IVDD progression, positioning lactylation-related genes and biomarkers as potential therapeutic targets. However, studies directly examining lactylation in IVDD remain limited. Shi et al. identified six hub lactylation-related genes as potential diagnostic markers, with chromobox protein homolog 3 (CBX3) notably upregulated in IVDD. Subsequent validation confirmed its relevance, and small-molecule inhibitors—particularly atosiban acetate, a CBX3 antagonist—were proposed for therapeutic use. Atosiban acetate prevented ECM degradation by increasing aggrecan (ACAN) and collagen type II alpha 1 chain (COL2A1) expression, while reducing matrix metalloproteinase 3 (MMP3) and a disintegrin and metalloproteinase with thrombospondin motifs 5 (ADAMTS5) levels, suggesting potential clinical benefit in IVDD. These findings highlight the pathogenic role of elevated lactylation in IVDD and identify atosiban acetate as a promising intervention [25]. Another study reported that reducing lactylation enhances matrix synthesis, promotes autophagy, and suppresses cellular senescence in NPCs. Notably, p300 expression remained unchanged in NPCs exposed to TNF-α, glutamine, or lactate, and no differences in p300 levels were observed between mildly and severely degenerated tissues, indicating that lactate—rather than p300—drives lactylation in NPCs. Glutamine exhibited effects similar to inhibitors of glycolysis and lactylation, suppressing glycolysis and regulating AMP-activated protein kinaseα (AMPKα) lactylation to influence matrix metabolism, senescence, and autophagy [69]. Moreover, protein lactylation was found to impact IVD vascularization as well as spliceosome and ribosome function. Collectively, these findings underscore the importance of lysine lactylation in regulating NPC metabolism, RNA splicing, and ribonucleoprotein complex biogenesis, thereby providing new insights into amino acid–specific protein lactylation under pathological normoxic conditions in IVDD [70].

Immune cell infiltration analysis in IVDD has revealed an increase in M0 macrophages and a decrease in follicular helper T cells [25]. Macrophage-mediated inflammation plays a pivotal role in modulating NPC function and viability. Infiltration of macrophages into degenerative IVD correlates with disease severity, elevated inflammatory cytokine levels, and ECM degradation, underscoring their indirect effects on NPCs [71]. Macrophage polarization further influences IVDD progression by regulating cell proliferation, inflammatory mediator secretion, and ECM homeostasis [72]. These effects are largely mediated through the release of pro-inflammatory factors and remodeling of the disc microenvironment. Notably, histone lactylation in macrophages can promote the transition from a pro-inflammatory to a homeostatic phenotype, thereby facilitating inflammation resolution [73,74]. Lactylation-related genes also exhibit strong associations with diverse immune cell populations, suggesting an important role in the immunometabolic landscape of IVDD. For example, insulin-like growth factor–binding protein 3 (IGFBP3), a lactylation-associated gene, showed a positive correlation with activated dendritic cells and negative correlations with activated CD8 T cells, NK cells, resting memory CD4 T cells, and memory B cells in IVDD [25]. These immunometabolic alterations likely contribute to remodeling of the disc microenvironment and disease progression.

In conclusion, the absence of a direct blood supply to the IVD creates a distinct microenvironment with low oxygen tension and restricted nutrient diffusion. As a result, resident cells such as NPCs and infiltrating macrophages rely predominantly on glycolysis for energy production, leading to lactate accumulation. While glycolysis initially drives pro-inflammatory gene expression, lactate subsequently acts as a critical modulator, exerting anti-inflammatory effects through histone lactylation. This dual role underscores the need for further research, particularly into the involvement of transcription factor EB–mediated autophagy in IVDD and the regulatory influence of lactate on autophagic pathways. Elucidating the interplay between lactate, histone lactylation, and inflammation resolution may provide new therapeutic opportunities for IVDD management.

## The lactylation in sarcopenia

Sarcopenia is an age-associated condition marked by progressive loss of skeletal muscle mass, strength, and functional performance. This decline contributes to increased morbidity, diminished quality of life, elevated mortality, and substantial healthcare costs. Globally, its prevalence among older adults is estimated at 10–16 %. The concept of *inflammaging*, first proposed by Franceschi et al. in 2000, describes the chronic, low-grade inflammation that accompanies immunosenescence during aging. Aging adipose tissue plays a pivotal role in this process, as immune cells—including macrophages and T lymphocytes—secrete chemokines that intensify inflammation within both skeletal muscle and adipose tissue. This sets in motion a self-perpetuating feedback loop: initial inflammation recruits additional immune cells, amplifying the inflammatory cascade and sustaining the chronic inflammatory state that characterizes aging.

Lactate, traditionally regarded as a mere byproduct of anaerobic metabolism, is now recognized as an important signaling molecule in skeletal muscle adaptation, particularly under mechanical overload. A review by Lawson et al. reports that elevated blood lactate concentrations during muscle overload are associated with activation of anabolic signaling pathways, implicating lactate in the promotion of muscle hypertrophy. In vitro evidence further suggests that lactate may upregulate myoblast markers and stimulate satellite cell proliferation, although findings remain inconsistent and the underlying mechanisms are not yet fully defined. Notably, lactate modulates histone lactylation—a post-translational modification sensitive to intracellular lactate levels—which appears to facilitate muscle regeneration. In C2C12 myoblasts, lactate administration increases histone lactylation, promotes myoblast differentiation, and enhances muscle regeneration, whereas inhibition of histone lactylation impairs myotube formation. Mechanistically, lactate-induced H3K9 lactylation has been shown to upregulate *Neu2*, a gene essential for myoblast differentiation.

Impaired muscle regeneration is a defining feature of sarcopenia. During muscle injury and repair, lactate serves not only as a metabolic byproduct but also as a key signaling molecule that modulates regeneration through epigenetic mechanisms. Immune cells function as both sensors and effectors of tissue damage; however, dysregulated immune responses can impede repair. After muscle injury, monocyte-derived Ly6C^hi macrophages adopt a pro-inflammatory phenotype, characterized by the upregulation of genes such as *Tlr4*, *Ifn1b*, *Il15*, and *Il6*, and the secretion of cytokines including TNF-α and IL-1β. These inflammatory macrophages stimulate muscle stem cell proliferation, trigger apoptosis in activated fibroadipogenic progenitors, and remove necrotic debris via efferocytosis. Subsequently, they transition into Ly6C^lo restorative macrophages that secrete growth factors such as insulin-like growth factor 1(IGF1), growth differentiation factor 3(GDF3), growth differentiation factor 15(GDF15), and transforming growth factor β (TGFβ), thereby promoting muscle progenitor cell differentiation, myofiber fusion, and extracellular matrix deposition. In parallel, these restorative macrophages enhance angiogenesis through the release of vascular endothelial growth factor (VEGF). This phenotypic transition is tightly regulated, with AMP-activated protein kinase (AMPK) acting as a critical mediator; AMPK deficiency disrupts this shift. Elevated glycolytic activity fuels inflammatory cytokine production and supports efferocytosis, whereas lactate-enriched conditioned media from efferocytic macrophages foster an anti-inflammatory milieu by activating anti-inflammatory gene programs in naïve macrophages. These findings position histone lactylation as a key epigenetic regulator of macrophage-mediated muscle regeneration.

Metabolic disorders are increasingly recognized as contributors to sarcopenia, with hyperglycemia potentially driving excessive lactate production and heightened skeletal muscle lactylation. Clinical studies report elevated fasting plasma lactate levels and increased skeletal muscle lactylation in obese individuals compared to lean counterparts. Consistent with in vivo findings, human skeletal muscle cells exposed to hyperglycemic conditions exhibit enhanced glycolytic flux, greater lactate accumulation, and correspondingly higher myotube lactylation. In contrast, treatment with 2-deoxy-D-glucose (2-DG)—an upstream glycolysis inhibitor—reduces both endogenous lactate levels and myotube lactylation. These results indicate that skeletal muscle lactylation is closely linked to glycolytic activity and lactate availability, paralleling patterns observed in other cell types. Given skeletal muscle’s central role in insulin sensitivity, and the association of lactylation with metabolic dysfunction, this modification may provide novel insight into the regulation of muscle metabolism, insulin resistance, and sarcopenia. Moreover, lactate and lactylation may function as signaling mediators in adaptive responses to exercise. In rodent models, high-intensity interval training (HIIT) elevates protein lactylation in the soleus muscle, liver, and adipose tissue within 24 h, with levels returning to baseline by 72 h. However, chronic resistance training that increases blood lactate and induces muscle hypertrophy over six weeks does not alter lactylation markers. These discrepancies likely reflect differences in exercise protocols and experimental design, underscoring the need for further investigation into the mechanisms and metabolic consequences of skeletal muscle lactylation.

Collectively, these findings support a significant role for lactylation in the pathogenesis of sarcopenia, potentially linking metabolic dysregulation, impaired muscle regeneration, and chronic inflammation.

## The dual roles of lactylation in tissue repair and inflammatory progression

Lactylation functions as a “double-edged sword” in inflammation and tissue remodeling, exerting either beneficial or detrimental effects depending on the specific context. The opposing pro-inflammatory and anti-inflammatory roles of this post-translational modification across various tissues reflect a complex interplay among metabolic, epigenetic, and immunological signals. Although lactylation is well recognized for promoting inflammation resolution and facilitating tissue repair, growing evidence indicates that it can also have harmful effects under pathological conditions. In MSDs such as OA, RA and IVDD, excessive or dysregulated lactate accumulation may lead to persistent histone lactylation, driving maladaptive gene expression [75].

A critical determinant of lactylation’s effects is cell-type specificity. In macrophages, histone lactylation frequently facilitates the phenotypic transition from pro-inflammatory M1 to anti-inflammatory M2 states, thereby promoting inflammation resolution and tissue regeneration. In contrast, in other cell types—such as fibroblasts, chondrocytes, and T cells—lactylation can enhance the expression of pro-inflammatory or matrix-degrading genes, driving pathological remodeling. This underscores that the functional outcome of lactylation is not universal but instead highly dependent on cellular identity and chromatin context. For instance, in OA, elevated histone H3K18la levels in both macrophages and chondrocytes have been linked to activation of the NF-κB signaling pathway and upregulation of MMPs, which directly contribute to cartilage degradation and ECM breakdown [76,77]. In this setting, rather than resolving inflammation, lactylation may reinforce catabolic activity and joint destruction. Similarly, in RA, lactate-induced lactylation affects not only macrophages but also synovial fibroblasts and T cells [78,79]. FLSs actively uptake lactate and undergo lactylation-associated activation, increasing their invasiveness and secretion of pro-inflammatory cytokines [28,80]. Concurrently, lactate promotes Th17 cell differentiation, thereby amplifying IL-17–mediated inflammation. In IVDD, macrophage-driven lactylation may sustain the release of inflammatory mediators that disrupt nucleus pulposus cell homeostasis and contribute to structural degeneration [81].

Second, the timing and duration of lactate exposure are critical determinants of its biological effects. Transient lactate accumulation, as occurs during acute inflammation or tissue repair, can induce adaptive lactylation that facilitates wound healing and promotes immune resolution [18,50]. By contrast, prolonged lactate overload, characteristic of chronic inflammatory states, may drive sustained lactylation at specific histone sites (e.g., H3K18la), thereby activating NF-κB–dependent transcriptional programs and amplifying inflammatory cascades [18].

Third, lactylation may interact with other epigenetic modifications, such as acetylation and methylation, producing either cooperative or antagonistic effects on transcription. For instance, competition for shared “writer” enzymes (e.g., p300) can alter the epigenetic landscape, potentially skewing gene expression toward pathogenic programs depending on the cell’s metabolic state [82,83].

In summary, the heterogeneous effects of lactylation across tissues and disease states result from the interplay of multiple factors, including cell type–specific regulatory landscapes, dynamic metabolic microenvironments, and epigenetic cross-regulation. Future studies should focus on delineating these interconnected mechanisms to enable precise modulation of lactylation pathways for therapeutic purposes while minimizing unintended effects.

## Integrating agonists or inhibitors of targeting lactylation molecules with SCLGI-resolving agents presents a novel strategy for treating MSDs

### Pharmaceutical treatment of MSDs

The pharmaceutical management of MSDs remains an unmet medical need, largely due to the complexity and uncertainty of their underlying pathogenesis. As a result, most current therapeutic approaches focus primarily on symptom relief, particularly pain management. Among MSDs, osteoporosis is distinctive in having a broad range of effective pharmacological agents capable of restoring impaired bone formation. These agents are generally classified into two main categories: antiresorptive and anabolic drugs. Antiresorptive agents, including bisphosphonates, selective estrogen receptor modulators (SERMs), and the anti-RANKL monoclonal antibody denosumab—which is notable for its relatively favorable safety profile—primarily function to inhibit bone resorption. However, due to the intrinsic physiological coupling between bone resorption and formation, they also inadvertently suppress bone formation. In parallel, anabolic strategies aim to stimulate bone-forming cells through agents such as human parathyroid hormone analogs (e.g., abaloparatide) and the anti-sclerostin monoclonal antibody romosozumab. Yet, these therapies also increase bone resorption as a consequence of the same physiological coupling. Consequently, both categories of osteoporosis treatments can lead to imbalances in bone remodeling in clinical settings [[84], [85], [86]].

For IVDD and OA, no effective disease-modifying pharmacological treatments have yet been established, and management remains largely palliative, relying primarily on non-steroidal anti-inflammatory drugs (NSAIDs). NSAIDs are also widely prescribed for RA. However, their long-term efficacy is limited, and their use is associated with increased risks of cardiovascular, cerebrovascular, gastrointestinal, and renal complications—particularly in elderly patients. Moreover, prolonged non-steroidal anti-inflammatory drug(NSAID) administration has been reported to exacerbate osteoporosis and sarcopenia. More targeted anti-inflammatory agents that inhibit key pro-inflammatory cytokines, such as TNF-α and IL-6, have shown short-term clinical benefits in patients with severe IVDD and RA. Nevertheless, these therapies carry substantial risks of serious adverse events, including recurrent infections and immunosuppression, which may facilitate tumor progression. Furthermore, discontinuation of such treatments often results in disease relapse, frequently in a more severe form [[87], [88], [89], [90]].

### Potential approaches for the discovery of novel lactylation agonists or inhibitors

The limitations of current pharmacological interventions for MSDs underscore the urgent need for innovative therapeutic strategies grounded in a comprehensive understanding of their underlying pathophysiology. This review emphasizes the emerging role of lactylation in the pathogenesis of IVDD, osteoporosis, OA, RA and sarcopenia, and proposes that targeting lactylation-related pathways may offer novel and more effective treatment avenues.

As outlined above, protein lactylation acts as a “double-edged sword” in human health, and its underlying mechanisms highlight lactylation modulation as a promising therapeutic strategy for tumors, metabolic disorders, and inflammation-related diseases. Preclinical studies provide strong support for this concept, and ongoing as well as future clinical trials are expected to further validate its therapeutic potential. A variety of small-molecule agonists and inhibitors targeting lactylation are currently under development, focusing on key processes such as lactate production, transport, receptor interaction, and regulation by epigenetic “writers” and “erasers” [24,91].

Targeting key glycolytic enzymes—such as LDHA, hexokinase (HK), pyruvate dehydrogenase kinase (PDK), PKM2, and PFKFB3—to regulate lactate production has emerged as a promising strategy for treating inflammation-associated diseases, with several candidate drugs progressing to clinical trials. For instance, Eriocitrin (ERI), a flavonoid, attenuates lung inflammation by inhibiting LDHA in a mouse model of acute lung injury [92]. Celastrol, a natural anti-inflammatory compound, interacts with LDHA to alleviate sepsis-induced tissue damage [93]. LDHA inhibitors such as gossypol and AT-101 have demonstrated notable efficacy in Phase II trials, both as monotherapies and in combination with standard chemoradiotherapy [94]. Stiripentol—an FDA-approved antiepileptic for Dravet syndrome—and its analogs inhibit LDH, thereby preventing the conversion of pyruvate to lactate while simultaneously conferring antiepileptic benefits [95]. The glycolytic inhibitor 2-DG has shown safety and efficacy in Phase I/II trials for prostate cancer and advanced solid tumors [96,97]. Notably, it crosses the blood–brain barrier via glucose transporters, suggesting potential applications in central nervous system disorders. In MK801-induced schizophrenia mouse models, 2-DG reduced lactate accumulation and H3K9 lactylation, resulting in improved behavioral outcomes [98]. Dichloroacetate (DCA) enhances mitochondrial glucose oxidation and lowers lactate levels by inhibiting PDK. Phase I trials have confirmed DCA’s feasibility and tolerability in patients with recurrent glioblastoma [99], while Phase II trials have demonstrated its safety when combined with chemoradiotherapy for advanced head and neck squamous cell carcinoma [100]. In AD research, PKM2 inhibitors such as shikonin disrupt the glycolysis/H4K12la/PKM2 feedback loop, thereby reducing lactate and H4K12la levels, inhibiting pro-inflammatory microglial activation, and improving learning and memory in AD mouse models [22]. Furthermore, the small-molecule inhibitor 3PO, which targets PFKFB3, can suppress H4K12la and attenuate excessive NF-κB–related gene transcription implicated in chronic kidney disease–associated fibrosis [23].

Targeting monocarboxylate transporters (MCTs) can effectively block lactate transport, and pharmacological inhibition of MCT4 has shown promise in attenuating disease progression through modulation of histone lactylation. For example, the MCT4 inhibitor VB124 significantly slows atherosclerosis progression in apolipoprotein E–deficient mice fed a high-fat diet by enhancing p300-mediated H3K18 lactylation, suggesting potential clinical applications in atherosclerosis therapy [101]. In addition, VB124 preserves cardiac function in type 2 diabetic mice, likely by modulating macrophage histone lactylation to suppress inflammation [102]. The dual MCT1/ monocarboxylate transporter2(MCT2) inhibitor AZD3965 has demonstrated favorable outcomes in patients with late-stage solid tumors and B-cell lymphoma in a Phase I clinical trial [103]. Furthermore, two ongoing clinical trials are evaluating the G protein–coupled receptor 81 (GPR81) inhibitor curcumin: one assessing its safety in children with acute lymphoblastic leukemia undergoing chemotherapy, and the other investigating its potential to delay or prevent disease progression in prostate cancer and other conditions when combined with piperine.

The formation and removal of lactylation are regulated by dedicated “writers” and “erasers,” offering novel therapeutic opportunities. The CREB-binding protein(CBP)/p300 inhibitor C646 attenuates inflammation in hepatic ischemia–reperfusion injury by suppressing HMGB1 lactylation [104], whereas A-485 exerts anti-angiogenic effects in proliferative retinopathy by targeting YY1 lactylation [105]. Histone deacetylase (HDAC) inhibitors such as MS-275 (entinostat) and apicidin increase H3K14la and H3K18ac levels but decrease H3K18la, likely due to crosstalk with acetylation, thereby downregulating the expression of fibrosis-associated genes [106,107]. Clinically approved HDAC inhibitors, including vorinostat and romidepsin, are already in use for lymphoma treatment [108]. In addition, SIRT3, which delactylates *CCNE2*, has emerged as a potential therapeutic target for liver cancer [109]. While targeted modulation of lactylation is promising, the fact that its “writers” and “erasers” are shared with other histone modifications—particularly acetylation—raises concerns about potential off-target effects. As mechanistic insights into lactylation in disease continue to expand, the development of more selective and precise therapeutic strategies is anticipated.

Although the roles of protein lactylation in health and disease have been increasingly documented, elucidating its specific molecular mechanisms remains essential for the rational design of targeted lactylation inhibitors and agonists with clinical applicability. Given its pivotal involvement in inflammation, a comprehensive mechanistic understanding of lactylation may provide a valuable foundation for the development of pharmacological agents capable of selectively activating or inhibiting this modification.

### Clinical applicability and limitations of Lactylation-Targeted therapies

While Table 1 identifies promising targets for modulating lactylation (e.g., LDHA, PKM2, MCTs, p300/CBP), their clinical translation requires careful evaluation of feasibility, druggability, and associated challenges. Several inhibitors, including LDHA inhibitors (e.g., gossypol, AT-101), PKM2 inhibitors (e.g., shikonin), and the PFKFB3 inhibitor 3PO, have shown efficacy in cancer and neurological models but are hindered by limitations such as poor tissue penetration, short half-lives, or dose-limiting toxicity. For example, gossypol’s clinical application is constrained by hepatotoxicity [110], whereas shikonin suffers from poor oral bioavailability [111]. Advances in nanoparticle-based delivery systems and tissue-specific prodrug design may help overcome these barriers and enhance their therapeutic potential [112].

**Table 1:** The role of lactate in each MSD.

Disease	Lactylation mechanism	Potential primary consequence	Potential therapeutic targets related to lactylation	Current gaps in knowledge	Current clinical evidence
Osteoporosis	Histone lactylation regulates macrophage polarization (M1 → M2) and promotes osteogenic differentiation; lactate-induced H3K18la in BMSCs modulates osteogenesis-related gene expression via p300; PKM2 and endothelial cell-derived lactate contribute to maintaining H3K18la levels; reduced H3K18la observed in OVX mice and patients with osteoporosis	(i) Anti-inflammatory effects via M2 polarization and suppression of IL-1β, IL-6, TNF-α; (ii) Promotion of osteogenesis and reduction of bone damage; (iii) Restoration of bone vessel density and improvement in bone parameters	LDHA; PKM2; p300; H3K18la; M1/M2 ratio	Detailed molecular pathways linking lactylation to bone remodeling remain incompletely understood; need for clinical validation of lactylation-targeted interventions; optimal delivery strategies and safety profiles remain unestablished	Human clinical sample evidence: Osteoporosis patients show reduced serum lactate and decreased H3K18la in BMSCs; related targets LDHA and PKM2 have small-molecule modulators under investigation in other diseases (e.g., oncology), indicating translational potential

OA	LDHA-mediated lactate production promotes histone lactylation (Pan-Kla, H3K18la) in chondrocytes, modulating inflammation, ROS generation, and cartilage matrix metabolism; LDHA knockdown reduces lactate levels, decreases histone lactylation, and preserves cartilage structure; lactate-induced histone lactylation facilitates M1-to-M2 macrophage polarization, potentially aiding tissue regeneration	(i) Reduction of cartilage degradation and MMP13 expression; (ii) Preservation of proteoglycan content and chondrocyte viability; (iii) Anti-inflammatory effects via M2 macrophage polarization	LDHA; H3K18la; histone acetyltransferase-like activity of LDHA; TPI1 locus	Direct mechanistic links between lactylation and OA progression remain limited; need for in vivo validation in human studies; lack of clinically approved lactylation-targeted OA therapies	No direct OA clinical trials; animal and in vitro models show increased Pan-Kla and H3K18la; LDHA inhibitors are in Phase I–II trials for cancer and metabolic disorders, indicating possible translational potential for OA

RA	Lactate accumulation in synovium promotes histone lysine lactylation in fibroblast-like synoviocytes (FLSs) via PI3K/Akt/NF-κB–mediated HIF-1α activation, enhancing glycolysis, migration, invasion, and IL-6 expression; artemisinin (ART) enhances p300–PKM2 interaction, increasing PKM2 lactylation and inhibiting nuclear translocation, thereby reducing FLS proliferation; lactate–lactylation axis modulates macrophage M2 polarization, impairs dendritic cell maturation, alters T cell migration and metabolism, and drives Th17 differentiation with increased IL-17 production; plasma cells with high lactylation scores show increased oxidative phosphorylation and glycolysis	(i) Pro-inflammatory activation of RA-FLSs; (ii) Enhanced inflammatory cytokine production (IL-6, IL-17); (iii) Promotion of Th17 differentiation; (iv) Regulation of macrophage polarization; (v) Potential modulation of plasma cell metabolism and autoantibody production	Histonp300; PKM2; HIF-1α; LDHA; MCT1; SLC5A12; NDUFB3; NGLY1; SLC25A4	Precise molecular mechanisms linking lactylation to immune cell function require further elucidation; lack of clinical validation in large patient cohorts; therapeutic targeting of lactylation pathways in RA remains untested in humans	Preclinical animal studies show ART-mediated PKM2 lactylation alleviates RA pathology; single-cell RNA-seq identifies lactylation-associated genes as potential biomarkers; PKM2 and p300 modulators under investigation in oncology and metabolic diseases indicate possible translational applicability for RA

IVDD	Glycolysis-dependent lactate accumulation in nucleus pulposus cells (NPCs) promotes histone lactylation, shifting metabolism toward catabolic pathways, inducing cellular senescence, ECM degradation, and modulating autophagy; CBX3 upregulation drives ECM breakdown; histone lactylation in macrophages promotes transition from pro-inflammatory to homeostatic phenotype; lactylation-related genes (e.g., IGFBP3) correlate with immune cell infiltration patterns	(i) ECM preservation via reduced MMP3 and ADAMTS5 expression; (ii) Increased ACAN and COL2A1 synthesis; (iii) Regulation of autophagy and senescence; (iv) Modulation of immune microenvironment through macrophage polarization	CBX3; atosiban acetate (CBX3 antagonist); AMPKα lactylation; glutamine as indirect lactylation regulator	Limited direct evidence on lactylation’s causal role in IVDD; unclear contribution of transcription factor EB–mediated autophagy; lack of in vivo human validation; mechanisms of immune cell–lactylation interactions remain to be clarified	Clinical tissue samples from IVDD patients show upregulation of CBX3; atosiban acetate validated in preclinical models to prevent ECM degradation; lactylation-related gene profiles correlated with immune cell infiltration patterns, suggesting translational potential

Sarcopenia	Lactate-induced histone lactylation (e.g., H3K9la) in skeletal muscle promotes myoblast differentiation and regeneration via Neu2 upregulation; lactylation in macrophages regulates phenotypic transition from pro-inflammatory Ly6C^hi to restorative Ly6C^lo state, enhancing myogenesis, ECM deposition, and angiogenesis; hyperglycemia increases skeletal muscle lactylation through enhanced glycolytic flux; exercise transiently elevates protein lactylation in skeletal muscle and other tissues	(i) Promotion of muscle regeneration and repair; (ii) Regulation of macrophage-mediated inflammation resolution; (iii) Potential influence on insulin sensitivity and metabolic homeostasis	H3K9la; Neu2; AMPK; glycolysis inhibitors (e.g., 2-DG)	Incomplete understanding of lactylation’s role in chronic vs. acute muscle adaptation; inconsistent findings across exercise models; lack of targeted interventions for sarcopenia; unclear long-term effects of modulating lactylation on muscle metabolism	Human studies show elevated fasting plasma lactate and increased skeletal muscle lactylation in obesity; hyperglycemia enhances lactylation in cultured human muscle cells; 2-DG reduces lactylation; rodent HIIT models confirm transient lactylation increase, suggesting potential translational relevance

Second, tissue-specific variability poses a significant challenge to the development of universally applicable therapies. In skeletal muscle, lactylation appears to facilitate tissue regeneration by promoting the macrophage-to-M2 transition, whereas in synovial tissue or cartilage, it may exacerbate pro-inflammatory signaling via the NF-κB pathway. These contrasting roles underscore the need for organ- or cell-specific delivery strategies and optimized dosing regimens, particularly when employing systemic modulators such as LDHA or MCT inhibitors.

Third, lactylation should be considered within the broader landscape of post-translational modifications (PTMs), including acetylation, methylation, crotonylation, and β-hydroxybutyrylation. While acetylation has been extensively characterized and therapeutically targeted—for example, through HDAC inhibitors—lactylation remains a comparatively novel and less-defined modification [113]. Nonetheless, it offers a distinctive metabolic connection between glycolytic flux and gene regulation, rendering it particularly relevant to MSDs, in which metabolic and inflammatory dysregulation frequently coexist. We propose that lactylation should be regarded not as a replacement for existing epigenetic targets, but as a complementary mechanism, with its specificity for lactate-driven pathologies providing a strong rationale for its prioritization in chronic inflammatory contexts. Despite the emerging promise of lactylation-targeted therapies, several critical limitations warrant consideration. First, the enzymes regulating lactylation—such as p300/CBP and HDACs—also catalyze other histone modifications (e.g., acetylation), creating the potential for widespread off-target epigenetic effects. For instance, p300 inhibition reduces both H3K18la and H3K18ac, complicating the attribution of observed phenotypes specifically to lactylation modulation [114].

Collectively, while delivery bottlenecks, tissue heterogeneity, and target selectivity remain formidable, emerging structure-guided chemistry, targeted degradation technologies, and organ-restricted delivery platforms now provide credible routes to clinical translation. Continued investment is warranted, particularly for combination strategies that simultaneously address metabolic and inflammatory dysregulation in MSDs.

### Bridging preclinical promise and human studies: Trial design considerations

To date, the therapeutic potential of lactylation-targeted drugs has been investigated exclusively in preclinical models, including cancer, sepsis, Alzheimer’s disease, and metabolic disorders. In MSDs such as OA and IVDD, no clinical trials have yet been initiated. We propose that early-phase clinical studies (Phase I/II) could be designed to assess the safety, tolerability, and preliminary efficacy of lactylation inhibitors or modulators in patients with moderate-to-severe OA or IVDD unresponsive to NSAIDs. For instance, a Phase I dose-escalation study could evaluate the safety of a locally administered LDHA inhibitor (e.g., via intra-articular injection) in patients with knee OA, followed by a Phase II randomized controlled trial measuring pain (WOMAC), joint space width, inflammatory biomarkers (e.g., IL-6, MMP-13), and synovial fluid lactate levels. Alternatively, oral MCT inhibitors (e.g., AZD3965) could be assessed in RA, using systemic endpoints. Incorporating biomarker-based patient stratification (e.g., baseline lactate or H3K18la levels) may help identify responsive subgroups. Exploratory endpoints could include MRI-based cartilage integrity assessments and quantification of histone lactylation in synovial biopsies.

### Resolution pharmacology in MSDs

The unresolved state of SCLGI is associated with the onset and persistence of various age-related disorders, including MSDs, suggesting that effective resolution of SCLGI could mitigate their symptomss [[2], [3], [4], [5], [6]]. The resolution of inflammation involves three sequential steps: suppression of pro-inflammatory signals, initiation of pro-resolving pathways, and clearance of dead cells and debris from the affected tissue. Mauro Perretti and colleagues introduced the term *resolution pharmacology* to describe the therapeutic use of SPMs, their small-molecule mimetics, and receptor agonists to treat chronic inflammatory conditions [115]. These agents restore the natural resolution of acute inflammation in cases where endogenous mechanisms are impaired.

Recent evidence indicates that nearly all SPM receptors are expressed on immune cells involved in inflammation resolution, including macrophages, T and B lymphocytes, neutrophils, and dendritic cells. Notably, key SPM receptors—particularly ALX/FPR2—are also expressed on musculoskeletal cell types such as chondrocytes, osteoclasts, fibroblasts, and skeletal muscle cells [[116], [117], [118], [119]]. This distribution suggests that SPM receptors contribute to inflammation resolution not only through immune cell modulation but also by directly targeting tissue-specific non-immune cells. Preclinical studies have consistently demonstrated that SPMs, especially resolvins and maresin 1, exert beneficial effects in arthropathies, osteoporosis, spinal disorders, and muscular dystrophy. Furthermore, clinical trials have shown that PUFAs, SPM precursors, improve outcomes in MSDs such as osteoarthritis, rheumatoid arthritis, osteoporosis, intervertebral disc degeneration, and sarcopenia [120]. Collectively, these findings support the therapeutic potential of pro-resolving agents for MSDs characterized by SCLGI.

The relationship between inflammation resolution and lactylation is an emerging area of investigation. As noted earlier, ART has been shown to alleviate RA symptoms by enhancing the interaction between p300 and PKM2, thereby promoting PKM2 lactylation. In AD models, PKM2 inhibitors such as shikonin disrupt the glycolysis/H4K12la/PKM2 feedback loop [22], leading to reduced lactate and H4K12la levels, suppression of microglial pro-inflammatory activation, and improved cognitive performance. These findings suggest that lactylation may mediate anti-inflammatory and protective effects in MSDs, highlighting its therapeutic potential. Recent evidence indicates that phosphatidic acids containing PUFAs selectively bind LDHA, inducing structural changes that inactivate the enzyme and function as negative LDHA modulators [121]. Notably, EPA treatment of human primary myotubes reduced pyruvate dehydrogenase kinase4 (PDK4) expression in cells derived from both obese and type 2 diabetes mellitus (T2DM) patients [122]. Although the underlying mechanisms remain incompletely understood, the insulin-sensitizing effects of omega-3 fatty acids may help restore glucose–fatty acid metabolic balance [123]. This aligns with prior findings that replacing dietary fat with omega-3 fatty acids prevented high-fat diet–induced increases in PDK4 expression in human skeletal muscle [124]. Furthermore, aspirin-triggered resolvin D1 has been reported to inhibit transforming growth factor β1 (TGF-β1)-induced epithelial-to-mesenchymal transition (EMT) by downregulating PKM2 via suppression of the mTOR pathway, a process associated with oxidative stress [125].

**Fig. 2:**
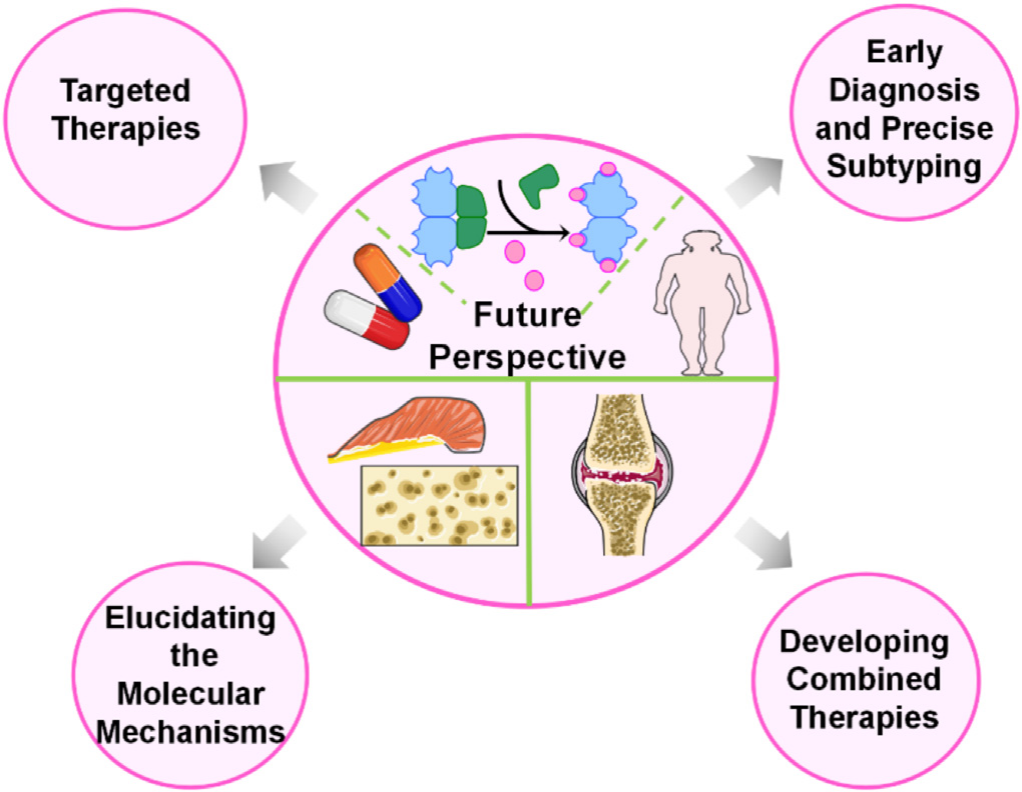
Future perspective A thorough understanding of the mechanisms behind musculoskeletal disorders (MSDs) and lactylation is crucial. Recent scientific and technological advancements enable us to assess these conditions at the tissue, cellular, and molecular levels. Timely and precise diagnosis is vital for the effective management of both MSDs and lactylation. Future research should focus on identifying and clinically applying new biomarkers for early detection. Advanced imaging techniques and molecular diagnostics can be employed to track disease progression and assess treatment responses. Given the complex factors involved in lactylation and MSDs, relying on a single treatment approach is often insufficient. Therefore, it is necessary to adopt a multifaceted treatment strategy that targets various pathways and cellular interactions. Combining agonists or inhibitors that focus on lactylation molecules with SCLGI-resolving agents represents an innovative approach to treating MSDs, potentially enhancing both the precision and efficacy of treatments. Ultimately, identifying effective targeted therapies, key regulatory factors, and early diagnostic methods will be essential for developing future treatments for MSDs.

The evidence presented here indicates that dysregulated lactylation and unresolved SCLGI are pivotal mechanisms driving the pathogenesis of IVDD, OA, RA and sarcopenia. These processes reinforce each other, creating a self-perpetuating cycle that accelerates MSD progression. We propose a novel therapeutic framework that combines modulation of lactylation with targeted resolution of SCLGI (Fig. 1a, Fig. 1b). Such agents reduce inflammation without impairing host defense, making them attractive and potentially safe candidates for MSD management. This approach underscores the value of promoting active resolution of inflammation rather than merely suppressing inflammatory signaling. While lactylation agonists, inhibitors, SPMs, and SPM receptor agonists are still in the early stages of clinical development, we advocate for their investigation as adjunctive anti-MSD therapies in combination with current treatment modalities (Fig. 2).

### A distinctive dual-target strategy: Integrating lactylation modulation with SCLGI resolution

Unlike conventional therapies for MSDs—such as NSAIDs and biologics—that primarily suppress inflammatory symptoms or inhibit individual cytokines, our review advocates a mechanistically distinct dual-target strategy: simultaneous modulation of lactylation and resolution of SCLGI. Rather than merely dampening inflammation, this approach aims to re-establish immune–metabolic homeostasis by addressing both the epigenetic regulation of immune cell plasticity (through lactylation) and the restoration of resolution-phase signaling via SPMs or GPCR agonists—pathways often deficient in chronic disease.

Whereas NSAIDs broadly inhibit cyclooxygenase (COX) enzymes and biologics such as TNF-α inhibitors block upstream cytokine signaling—often at the expense of host defense and timely tissue repair—lactylation-targeted interventions aim to reprogram immune cells toward a pro-resolving, reparative phenotype without inducing global immunosuppression. In parallel, agents that resolve SCLGI do not simply suppress inflammatory processes but instead facilitate their active resolution, a mechanistically distinct and physiologically conserved pathway. The combined use of these two strategies holds the potential to synergistically repolarize macrophages, restore immune–microenvironmental balance, and reinstate tissue repair programs more effectively than either approach alone.

This integrative model offers several advantages: (1) it addresses both the metabolic drivers and the inflammatory persistence underlying MSDs; (2) it has the potential to reduce reliance on prolonged immunosuppressive therapy; and (3) it is adaptable to different stages of disease progression. By prioritizing a systems-level intervention, this approach may disrupt the self-perpetuating cycle of chronic inflammation and tissue degeneration that defines many MSDs.

## Future perspectives: translational challenges, biomarker development, and clinical pathways

(1) Translational challenges

The clinical translation of lactylation-targeted therapies for MSDs is hindered by several major challenges. First, tissue-specific lactylation dynamics complicate therapeutic development: in skeletal muscle, lactylation appears to promote regeneration through macrophage polarization, whereas in synovium or degenerative intervertebral discs, it may potentiate NF-κB–mediated inflammation and extracellular matrix degradation. Second, currently available lactylation modulators, such as p300 inhibitors, lack target specificity and frequently affect other post-translational modifications (e.g., acetylation), thereby increasing the risk of off-target effects. Third, the spatiotemporal regulation of lactylation in vivo remains insufficiently understood, owing to technical constraints in metabolic imaging and epigenetic profiling. Addressing these barriers will require the development of organ-specific drug delivery platforms, highly selective modulators, and advanced molecular imaging technologies.

(2) Biomarker development for early detection and monitoring

Early diagnosis and treatment monitoring will be essential for the successful implementation of lactylation-based interventions. Promising biomarker strategies include:

• Molecular markers: quantification of lactate concentrations, histone lactylation marks (e.g., H3K18la), and lactylation-related gene expression in synovial fluid, serum, or tissue biopsies;
• Metabolic profiling: integration of metabolomic signatures with lactate flux measurements to identify patients most likely to benefit from lactylation modulators;
• Imaging biomarkers: application of advanced modalities such as MRI, CBCT, or PET tracers to evaluate both structural alterations and tissue-specific metabolic activity.

Combining these complementary approaches may facilitate personalized therapy selection and enable longitudinal monitoring of therapeutic efficacy.

(3) Future clinical directions

Given the multifactorial pathogenesis of MSDs, multimodal treatment strategies are likely to be more effective than single-agent interventions. We propose combining lactylation agonists or inhibitors with SCLGI-resolving agents, targeted physical rehabilitation, and nutritional measures such as omega-3 polyunsaturated fatty acid (PUFA) supplementation to synergistically enhance tissue repair and modulate inflammation. Precision medicine approaches should incorporate genomic profiling (e.g., MCT transporter or HIF-1α variants) and metabolic monitoring to optimize patient selection. Early-phase clinical trials could evaluate targeted delivery of LDHA or MCT inhibitors in OA or IVDD, with endpoints including biomarker modulation and imaging-based structural preservation.

## Concluding remarks

Protein lactylation represents a “double-edged sword” in human health. On one hand, it supports essential physiological processes, including osteoblast differentiation, bone formation, transcriptional elongation of embryo-related genes, DNA repair, and the amelioration of fatty liver. On the other, excessive lactylation can aggravate inflammation and MSDs. Metabolic shifts—particularly lactate accumulation during chronic inflammation—may dysregulate histone lactylation, triggering pathogenic pathways that drive chronic inflammatory diseases. This review critically examines both the beneficial and detrimental aspects of lactylation. Although its physiological roles are increasingly recognized, further studies are needed to clarify the underlying molecular mechanisms, enabling the development of targeted lactylation modulators for clinical use.

Our review evaluates the hypothesis that dysregulated lactylation and impaired resolution of SCLGI are central drivers in the pathogenesis of IVDD, osteoporosis, OA, RA, and sarcopenia. We propose a complementary therapeutic strategy that combines lactylation agonists or inhibitors with agents that actively promote the resolution of chronic inflammation. Realizing this approach, however, will require overcoming several critical research and translational challenges.

### Outstanding questions

Key questions remain: Are the relationships between lactylation, SCLGI, and MSDs causal or independent? Would the use of lactylation agonists or inhibitors, in conjunction with SPM mimetics and receptor agonists, be effective for patients with MSDs? Furthermore, does the combined use of these agents enhance treatment efficacy compared to their individual application? More research is needed to clarify the specificity, safety, tolerability, side effects, and overall effectiveness of these compounds. Additionally, we must ascertain at what stage of these disorders lactylation becomes dysregulated, and whether patients with MSDs can be subtyped based on lactylation signaling profiles or SCLGI levels. Identifying these profiles may provide valuable insights for patient stratification and a deeper understanding of disease mechanisms (Fig. 2). If our hypothesis is validated, it would support the use of lactylation modulators and SPM analogs as a novel treatment strategy, potentially improving relief from the pathological manifestations of these disorders.

### Search strategy and selection criteria

Literatures published between 2012 and 2025 were obtained by searches of PubMed and Web of Sciences using the search terms “lactylation”, “osteoporosis”, “osteoarthritis”, “sarcopenia”, “intervertebral disc degeneration intervertebral disc degeneration” and “rheumatoid arthritis”alone or in combinations. Abstracts and reports from meetings were included only when they related directly to previously published work. Only original research papers published in English were included.

## Consent for publication

Not applicable.

## Funding declaration

Supported by the National Natural Science Foundation of China (82102656 to Tiantian Wang, 82471388 to Zhen Hong) and the 1.3.5 Project for Disciplines of Excellence, West China Hospital, Sichuan University (ZYGD23032).

## Compliance with ethics requirements

This article does not contain any studies with human or animal subjects.

## CRediT authorship contribution statement

**Tiantian Wang:** Conceptualization, Writing – original draft, Writing – review & editing. **Sihan Chen:** Writing – review & editing. **Zhen Hong:** Conceptualization, Writing – review & editing.

## Declaration of competing interest

The authors declare that they have no known competing financial interests or personal relationships that could have appeared to influence the work reported in this paper.
